# Clinical features and risk factors for postoperative recurrence in patients with Cushing's syndrome of different etiologies

**DOI:** 10.1038/s41598-024-53913-4

**Published:** 2024-02-26

**Authors:** Yunjia Cai, Xue Zhao, Linan Ren, Siyuan Liu, Xinming Liu, Xiaokun Gang, Guixia Wang

**Affiliations:** Department of Endocrinology and Metabolism, The First Hospital of Jilin University, 71 Xinmin Street, Chaoyang District, Changchun, 130021 Jilin People’s Republic of China

**Keywords:** Cushing’s syndrome, Cushing’s disease, Adrenal adenoma, Ectopic ACTH syndrome, Clinical features, Recurrence, Endocrinology, Endocrine system and metabolic diseases

## Abstract

The clinical characteristics of Cushing’s syndrome (CS) vary with etiology, and few studies have investigated the risk factors affecting CS recurrence after surgery. This retrospective study involved 202 patients diagnosed with CS between December 2012 and December 2022. The patients were divided into three groups according to etiology: Cushing's disease (CD), adrenocortical adenoma (ACA), and ectopic adrenocorticotropic hormone (ACTH) syndrome (EAS). Of the patients with CS, 41.9% had hypokalemia and 15.0% had hypophosphatemia. The cortisol levels were negatively correlated with blood potassium, blood chlorine, and blood phosphorus. Moreover, 22.4% of patients had an abnormal heart structure, 11.2% had centripetal remodeling, 5.6% had centripetal hypertrophy, and 5.6% had centrifugal hypertrophy. The overall recurrence rate of CS caused by pituitary tumors and adrenal adenoma was 25.7%. The recurrence times were longer in the ACA group versus the CD group, in patients < 50 years of age versus in patients ≥ 50 years old group, and in patients with CD with tumors ≥ 1 cm versus tumors < 1 cm. Age, preoperative cortisol level, postoperative cortisol level, and absolute neutrophil value were closely related to postoperative recurrence, and etiology was an independent predictor of tumor recurrence in patients with CS. The results of this study showed that CS caused by different etiologies showed different clinical manifestations, blood electrolyte characteristics, and that CS could affect patient cardiac structure and function. Etiology is an independent predictor of tumor recurrence in patients with CS.

## Introduction

Cushing's syndrome (CS) is an endocrine disorder caused by high levels of cortisol in the body, and includes both endogenous and exogenous CS. Exogenous CS, caused by long-term use of medications containing corticosteroids, is the most common cause. Endogenous CS is caused by the body's own production of excess cortisol. The annual incidence of CS is 2–3 per million people^1,2^. Endogenous CS can be classified as adrenocorticotropic hormone (ACTH)-dependent and non-ACTH-dependent, occurring in approximately 80% and 20% of cases, respectively. In ACTH-dependent types, the ratio of Cushing disease to ectopic ACTH syndrome is approximately 7:1^3,4^. The clinical manifestations of CS vary; the common clinical features include central obesity, hypertension, dyslipidemia, type 2 diabetes, osteoporosis, hirsutism, menstrual disorders, and abnormal mental function^5^. Hypokalemia is a common biochemical alteration in patients with CS and is more common in patients with ectopic ACTH syndrome than in those with Cushing's disease^6^. One study reported that 16% of patients with CS had hypophosphatemia. Twenty-four-hour urinary free cortisol (24H-UFC) levels are negatively correlated with serum phosphate concentration, and serum phosphate levels increase after CS remission^7^.

Chronic hypercortisolism causes structural and functional changes in the heart. Up to 70% of patients with active CS have abnormal left ventricular mass parameters, with 42% showing centripetal hypertrophy and 23% showing ventricular remodeling. When CS is cured or in remission, the left ventricular mass parameters improve significantly but are not completely normal^8^. Peter et al. showed that in the absence of dense myocardial fibrosis, patients with CS often have significant subclinical biventricular and left atrial systolic dysfunction and an increased left ventricular mass. Successful treatment of CS improves ventricular and atrial systolic function and reduces left ventricular mass and myocardial thickness, while improving glucose metabolism and body mass index (BMI)^9^. To our knowledge, this is the first study to analyze the influence of different etiologies on cardiac structural function.

The postoperative recurrence rate of CS differs according to etiology. Long-term follow-up studies have shown a significantly lower survival rate of patients who relapse after surgery is significantly lower than that of patients in remission. Therefore, studying the factors associated with relapse is important. However, few studies have analyzed the risk factors for relapse. The present study included 202 patients with CS and analyzed and compared the clinical characteristics of CS patients according to the different etiologies to improve the accuracy of clinical classification diagnosis. This study also explored the risk factors affecting CS recurrence after surgery and is the first to propose etiology as an independent predictor of CS recurrence. This study provides a basis for early clinical intervention to prevent disease recurrence.

## Materials and methods

### Participants

We reviewed the clinical data of 202 patients diagnosed with CS at First Hospital of Jilin University between December 2012 and December 2022. Demographic information was collected at baseline, including data on sex; age; height; weight; BMI; systolic blood pressure; diastolic blood pressure; disease course; clinical features; first diagnosis symptoms; ions; sex hormones; cardiac structure and function; and serum cortisol, ACTH, and 24H-UFC levels. They were divided into the adrenocortical adenoma (ACA), Cushing's disease (CD) (pituitary tumor), and ectopic ACTH syndrome (EAS) (ectopic adrenocorticotropin tumor) groups according to the different tumor sources. CS recurrence was defined as the reappearance of clinical symptoms and biochemical features caused by hypercortisolemia after the remission period. We followed up all patients with CS caused by pituitary tumors and adrenal adenomas and recorded the time of tumor recurrence, which was the endpoint of follow-up. For those with no recurrence and those lost at the follow-up endpoint, it was defined as a deletion event.

### Inclusion and exclusion criteria

The inclusion criteria were: (1) Patients diagnosed with CS according to the Endocrine Society clinical practice guidelines and confirmed by postoperative pathology in all patients who underwent surgical resection. Diagnostic criteria for CS: elevated 24-h UFC levels (performed at least 2 times), and serum cortisol levels ≥ 50 nmol/L after an overnight 1-mg dexamethasone suppression test. (2) The etiology of CS has been clearly defined: After the diagnosis of CS was confirmed, the ACTH concentration was first measured, and if the ACTH concentration was < 10 pg/mL suggesting non-ACTH-dependent CS, further adrenal tumors seen on adrenal CT or MRI were diagnosed as ACA. ACTH-dependent CS was considered if ACTH was > 20 pg/mL. CD was diagnosed if cortisol levels were suppressed (reduced by > 50%) after a high-dose dexamethasone suppression test (HDDST), cortisol levels increased after DDAVP or CRH stimulation, and pituitary MRI showed a pituitary tumor. EAS was diagnosed if cortisol levels were not suppressed after the HDDST test, there was no response to DDAVP or CRH stimulation, and an ectopic lesion was found on CT or MRI. For patients with ACTH in the range of 10–20 pg/mL, reassessment is warranted. ACTH-dependent CS should be considered if accompanied by typical clinical symptoms.

The exclusion criteria were: (1) long-term use of exogenous adrenal corticosteroids. (2) long-term heavy drinking (≥ 30 g/day for men and 15 g/day for women). (3) false CS due to depression or obesity (patients who have clinical symptoms similar to those of CS but are able to rule out CS by tests such as the LDDST). (4) patients with severe organ function impairment.

### Correlation variable definitions

(1) BMI = weight (kg)/height (m)^2^. (2) neutrophil-to-lymphocyte ratio (NLR) = neutrophil absolute value/lymphocyte absolute value; (3) E/A ratio = left ventricular early diastolic rapid filling peak/late diastolic filling peak; (4) left ventricular mass index (LVMI) = left ventricular mass (LVM)/body surface area (BSA); LVM (g) = 0.8 * 1.04 * [(end diastolic septal thickness + left ventricular posterior wall thickness + left ventricular end-diastolic diameter)^3^ − left ventricular end-diastolic diameter^3^] + 0.6 (5) Relative wall thickness (RWT) = 2 * left ventricular posterior wall thickness/left ventricular end-diastolic diameter.

### Assay characteristics

Normal reference ranges: blood glucose 3.9–6.1 mmol/L, potassium 3.5–5.3 mmol/L, sodium 137–147 mmol/L, chlorine 99–110 mmol/L, calcium 2.11–2.52 mmol/L, phosphorus 0.80–1.45 mmol/L, LVMI > 125 g/m2 (men) and > 120 g/m2 (women), RWT < 0.45.

Hypertension is defined as a systolic blood pressure ≥ 140 mmHg and/or a diastolic blood pressure ≥ 90 mmHg for all three blood pressure measurements. Diabetes mellitus is defined as diabetes mellitus with symptoms + random blood glucose level ≥ 11.1 mmol/L or a fasting blood glucose level of ≥ 7.0 mmol/L, or 2-h blood glucose level ≥ 11.1 mmol/L in a glucose tolerance test (two episodes of intravenous blood glucose). Potassium levels < 3.5 mmol/L were defined as hypokalemia. Phosphorus levels < 0.8 mmol/L were defined as hypophosphatemia.

### Statistical analysis

The measurement data are described as means ± standard deviations if they conformed to a normal distribution and as medians and interquartile distances otherwise. Counting data are described as frequencies and percentages. If the numerical variables conformed to a normal distribution, a comparison of multiple sample means was performed using a one-way analysis of variance (ANOVA). If there a difference in the overall comparison of multiple groups was identified, further pairwise comparisons were performed. If the numerical variables did not conform to a normal distribution, the Wilcoxon signed-rank sum test was applied, and the Kruskal–Wallis H test was used to compare multiple groups of independent samples. Data were compared using Pearson’s chi-squared test. Spearman’s correlation analysis was used to analyze two variables. Survival analysis was performed using the Kaplan–Meier method. The Cox proportional hazards model was used to analyze the risk factors for tumor recurrence in patients with CS. Statistical significance was set at P < 0.05. IBM SPSS Statistics for Windows, version 26.0 (IBM Corp., Armonk, NY, USA) and R version 4.2.3 (The R Foundation for Statistical Computing, Vienna, Austria) were used to perform the statistical analyses.

### Statement

This study complied with the principles of the Declaration of Helsinki and was approved by the Ethics Committee of the First Hospital of Jilin University. All authors confirmed that all methods were carried out in accordance with the relevant guidelines and regulations. All data used in this study were anonymized and the requirement for informed consent was waived.

## Results

### Baseline characteristics

Our study included 202 patients with CS who were divided into the CD (122 cases, 60.40%), ACA (71 cases, 35.15%), and EAS (9 cases, 4.45%) groups according to the different etiologies. Regarding sex, 176 patients were female (87.1%) and 26 were male (12.9%). Women accounted for 95.8%, 84.4%, and 55.6% of patients in the ACA, CD, and EAS groups, respectively. The proportions of women in the CD and ACA groups were higher than that in the EAS group. The median age at CS onset was 46 (32, 54) years, including 42 (30, 51) years in the ACA group and 56 (48.5, 67.5) years in the EAS group. Regarding blood pressure, the 145 patients with hypertension accounted for 71.78% of the total population. The median course of disease in patients with CS was 12 (12, 36) months. The median disease duration was 18 (12, 36) months in the CD group and 3.5 (1, 10.75) months in the EAS group (Table 1).

**Table 1:** Baseline characteristics of patients with CS with different etiologies.

	Total (N = 202)	ACA (N = 71)	CD (N = 122)	EAS (N = 9)	P value
Sex (female)	176 (87.1%)	68 (95.8%)^a^	103 (84.4%)	5 (55.6%)^a^	0.001*
Age (years)	46 (32, 54)	42 (30, 51)^a,b^	47 (34, 56)^a^	56 (48.5, 67.5)^b^	0.001*
Height (cm)	161 (160, 166.25)	160 (158, 165)^a^	162 (160, 166)	172 (166.5, 182)^a^	0.015*
Weight (kg)	67.5 (60, 77.125)	65 (60, 75)	70 (55, 80)	70 (58.75, 77.50)	0.755
BMI (kg/m^2^)	25.7 (22.96, 27.97)	25.78 (22.86, 27.84)	26.05 (23.44, 29.22)	23.66 (22.58, 25.90)	0.345
Systolic pressure (mmHg)	142 (130, 159.5)	140 (130, 160)	143 (130.00, 154.25)	160 (144, 171.5)	0.058
Diastolic pressure (mmHg)	90 (80, 101)	90 (80, 102.25)	89 (80, 100)	97 (91.5, 115.5)	0.128
Time to diagnose (months)	12 (12, 36)	12 (12, 30)^a^	18 (12, 36)^b^	3.5 (1, 10.75)^a,b^	0.003*

*is P < 0.05, which has statistical significance. a, b, and c indicate that the pairwise comparison is statistically significant.

### Clinical manifestation

Clinical manifestations of CS differ among patients with different etiologies. In the ACA group, 81.69% of patients showed central obesity, which was the most common clinical feature, followed by hypertension, purple skin lines, fatigue, and edema, whereas infection and acne were relatively rare. In the CD group, hypertension was the most common clinical feature, accounting for 67.21% of the cases, followed by central obesity, diabetes, fracture, and facial plethora, whereas infection and acne were relatively rare. In the EAS group, hypertension was the most common clinical symptom (88.89%), followed by edema, diabetes, fatigue, and infection, whereas menstrual disorders were relatively rare.

**Figure 1:**
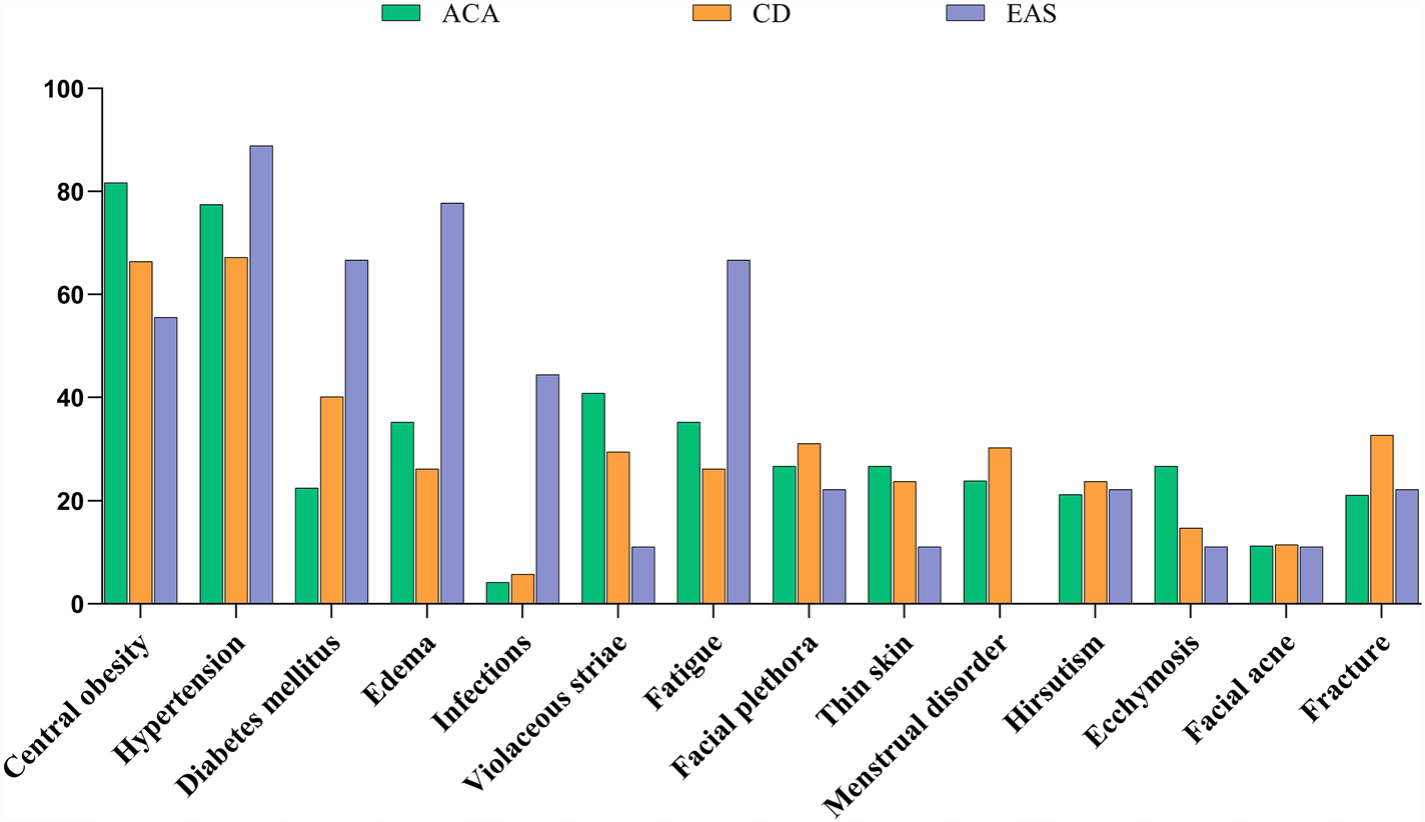
Symptom distribution in patients with Cushing’s syndrome (%). ACA, adrenocortical adenoma; CD, Cushing’s disease; EAS, ectopic adrenocorticotropic hormone (ACTH) syndrome.

In the EAS group, 66.67% of patients had diabetes, a proportion that differed significantly different from those in the ACA (22.54%) and CD (40.16%) groups (P < 0.05). Among the patients in the EAS group, 77.78% had edema, a significantly higher proportion compared to those in the ACA (35.21%) and CD (26.23%) groups (P < 0.05). In the EAS group, 44.44% of patients had an infection, which was statistically significant compared with the ACA (4.23%) and CD (5.74%) groups (P < 0.05). The most common manifestations of CS overall were hypertension (71.78%), central obesity (71.29%), and diabetes (35.15%). Infection and acne were relatively rare among all common symptoms, accounting for 6.93% and 11.39% of cases, respectively (Table 2 and Fig. 1).

**Table 2:** Comparison of clinical manifestations of patients with Cushing’s syndrome in different etiological groups.

	Total (N = 202)	ACA (N = 71)	CD (N = 122)	EAS (N = 9)	P value
Central obesity	144 (71.29%)	58 (81.69%)^a^	81 (66.39%)	5 (55.56%)^a^	0.044*
Hypertension	145 (71.78%)	55 (77.46%)	82 (67.21%)	8 (88.89%)	0.158
Diabetes mellitus	71 (35.15%)	16 (22.54%)^a,b^	49 (40.16%)^a^	6 (66.67%)^b^	0.006*
Edema	64 (31.68%)	25 (35.21%)^a^	32 (26.23%)^b^	7 (77.78%)^a,b^	0.004*
Infections	14 (6.93%)	3 (4.23%)^a^	7 (5.74%)^b^	4 (44.44%)^a,b^	0.000*
Violaceous striae	66 (32.67%)	29 (40.85%)	36 (29.51%)	1 (11.11%)	0.100
Fatigue	63 (31.19%)	25 (35.21%)	32 (26.23%)^a^	6 (66.67%)^a^	0.027*
Facial plethora	59 (29.21%)	19 (26.76%)	38 (31.15%)	2 (22.22%)	0.726
Thin skin	49 (24.26%)	19 (26.76%)	29 (23.77%)	1 (11.11%)	0.576
Menstrual disorder	54 (26.73%)	17 (23.94%)	37 (30.33%)	0 (0%)	0.112
Hirsutism	46 (22.77%)	15 (21.27%)	29 (23.77%)	2 (22.22%)	0.914
Ecchymosis	38 (18.81%)	19 (26.76%)	18 (14.75%)	1 (11.11%)	0.100
Facial acne	23 (11.39%)	8 (11.27%)	14 (11.48%)	1 (11.11%)	0.999
Fracture	57 (28.22%)	15 (21.13%)	40 (32.79%)	2 (22.22%)	0.204

### First symptoms

**Figure 2:**
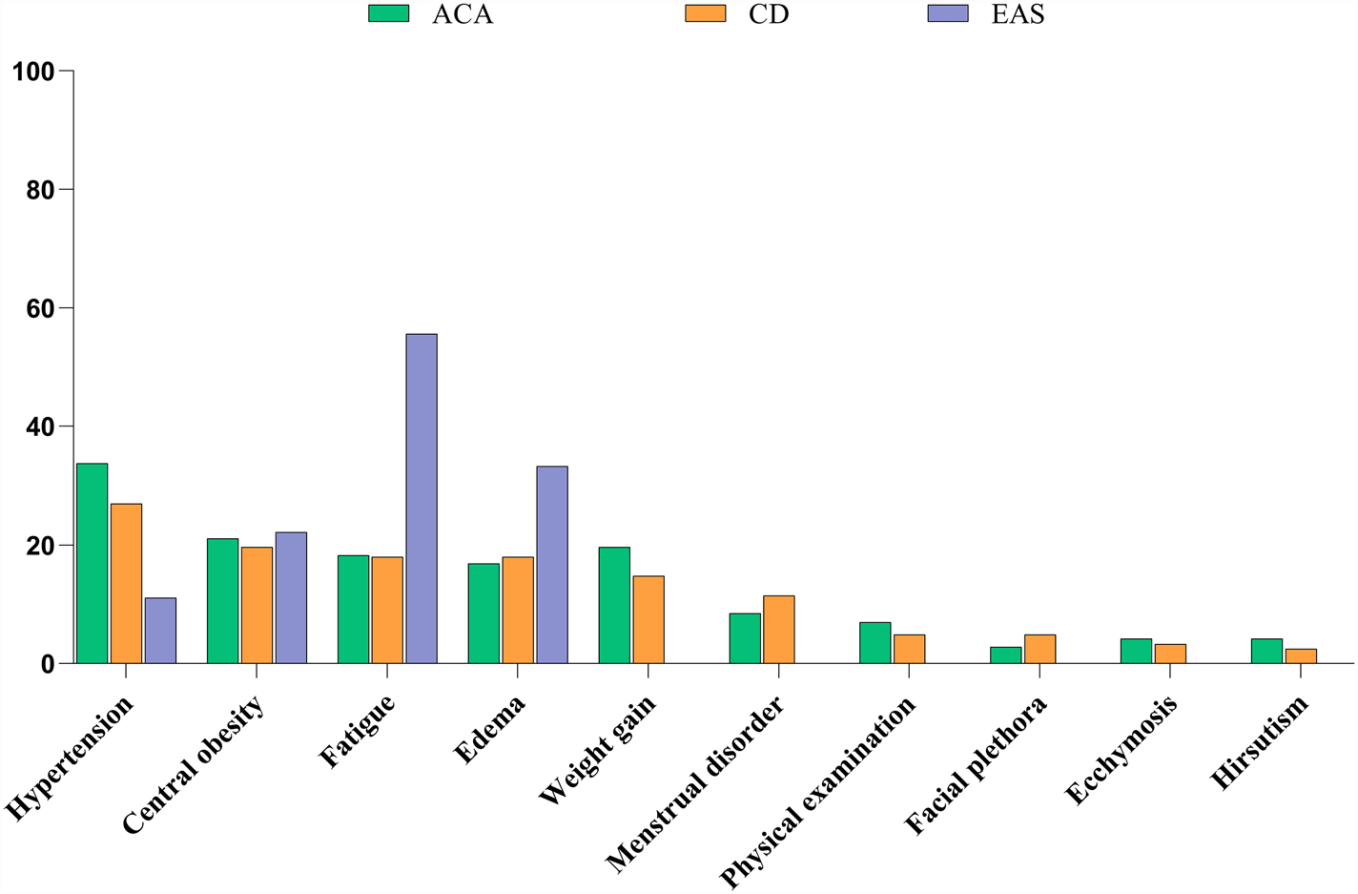
Distribution of the first diagnosis symptoms in patients with Cushing’s syndrome (%). ACA, adrenocortical adenoma; CD, Cushing’s disease; EAS, ectopic adrenocorticotropic hormone (ACTH) syndrome.

In the study population, hypertension (28.7%) was the most common first diagnostic symptom, followed by central obesity (20.3%), fatigue (19.8%), and edema (18.3). In the ACA group, hypertension (33.8%) was the most common initial diagnostic symptom, followed by central obesity (21.1%), weight gain (19.7%), and fatigue (18.3%). In the CD group, hypertension (27.0%) was the most common first diagnostic symptom, followed by central obesity (19.7%), fatigue (18.0%), and edema (18.0%). In the EAS group, fatigue (55.6%) was the most common initial diagnostic symptom, followed by edema (33.3%), central obesity (22.2%), and hypertension (11.1%) (Table 3 and Fig. 2).

**Table 3:** Comparison of first diagnosis symptoms among different etiological groups in patients with Cushing’s syndrome (CS).

	Total (N = 202)	ACA (N = 71)	CD (N = 122)	EAS (N = 9)	P value
Hypertension	58 (28.7%)	24 (33.8%)	33 (27.0%)	1 (11.1%)	0.297
Central obesity	41 (20.3%)	15 (21.1%)	24 (19.7%)	2 (22.2%)	0.961
Fatigue	40 (19.8%)	13 (18.3%)^a^	22 (18.0%)^b^	5 (55.6%)^a,b^	0.023*
Edema	37 (18.3%)	12 (16.9%)	22 (18.0%)	3 (33.3%)	0.482
Weight gain	32 (15.8%)	14 (19.7%)	18 (14.8%)	0 (0%)	0.272
Menstrual disorder	20 (9.9%)	6 (8.5%)	14 (11.5%)	0 (0%)	0.305
Physical examination	11 (5.4%)	5 (7.0%)	6 (4.9%)	0 (0%)	0.497
Facial plethora	8 (4.0%)	2 (2.8%)	6 (4.9%)	0 (0%)	0.529
Ecchymosis	7 (3.5%)	3 (4.2%)	4 (3.3%)	0 (0%)	0.683
Hirsutism	6 (3.0%)	3 (4.2%)	3 (2.5%)	0 (0%)	0.605

### Blood electrolyte levels

**Figure 3:**
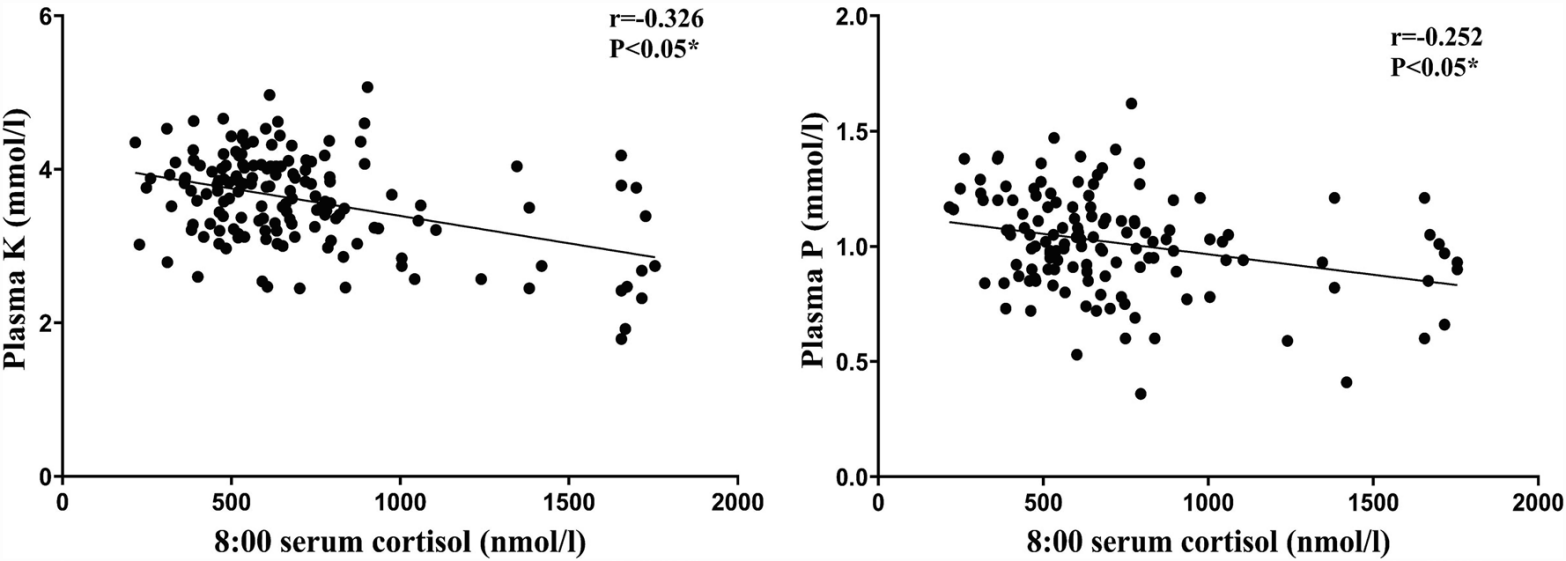
Serum cortisol concentrations at 8:00 are negatively correlated with serum potassium (r = − 0.326, P < 0.05) and serum phosphorus (r = − 0.252, P < 0.05) concentrations.

Of the 202 patients with CS, 41.9% had hypokalemia, including 32.8%, 43.7%, and 100% of patients in the ACA, CD, and EAS groups, respectively. Hypophosphatemia was present in 15.0% of patients, including 15.8%, 18.7%, and 28.6% of patients in the ACA, CD, and EAS groups, respectively (Table 4). The results of the correlation analysis showed that serum cortisol and urinary free cortisol levels were negatively correlated with serum potassium, blood chlorine, and blood phosphorus levels (P < 0.05) (Fig. 3).

**Table 4:** Comparison of electrolyte characteristics among different etiological groups in patients with Cushing’s syndrome.

	Total	ACA	CD	EAS	P value
K	3.57 ± 0.62	3.80 ± 0.54^a^	3.51 ± 0.56^b^	2.52 ± 0.42^a,b^	0.000*
Na	143.42 ± 3.15	143.33 ± 2.53	143.31 ± 3.29	145.05 ± 5.27	0.228
Cl	104.12 ± 3.74	104.83 ± 3.47^a^	104.23 ± 3.37^b^	98.06 ± 3.94^a,b^	0.000*
Ca	2.18 ± 0.15	2.22 ± 0.15^a^	2.17 ± 0.14^b^	2.02 ± 0.18^a,b^	0.001*
P	1.02 (0.89, 1.17)	1.04 (0.90, 1.17)	0.99 (0.86, 1.19)	0.98 (0.71, 1.26)	0.500

K, potassium; Na, sodium; Cl, chloride; Ca, calcium; P, phosphorus.

### Cardiac structure and function

**Figure 4:**
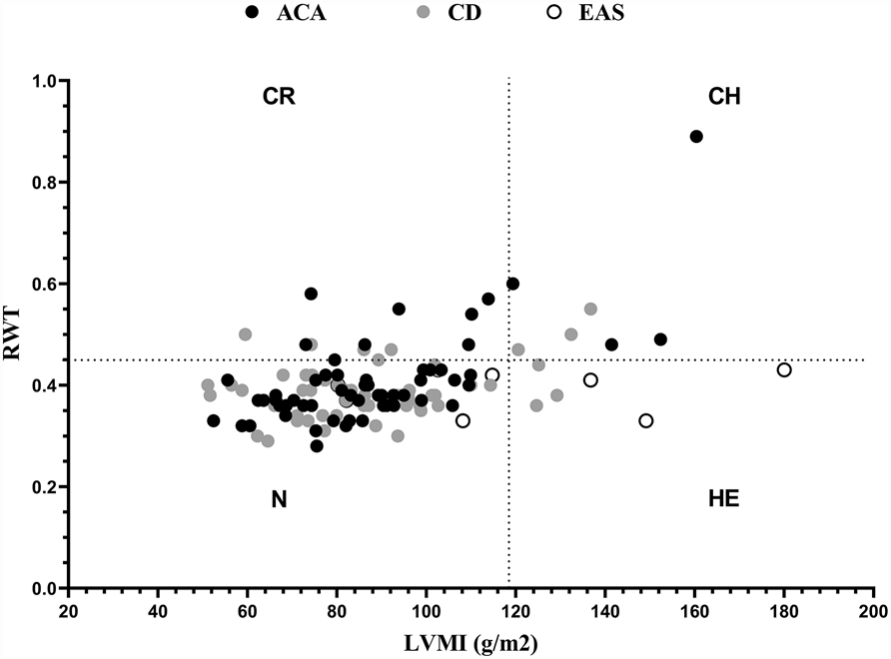
Cardiac structure of patients with Cushing’s syndrome analyzed by Ganau method. LVMI: left ventricular mass index; RWT, relative wall thickness; ACA, adenocarcinoma; CD, Cushing’s disease; CR, concentric remodeling; HE, eccentric hypertrophy; CH, centripetal hypertrophy.

Troponin, creatine kinase isoenzyme (CK-MB), pro-brain natriuretic peptide (pro-BNP), right ventricular diameter, interventricular septal thickness, left ventricular end-diastolic diameter, and left ventricular mass index differed significantly among the three groups (P < 0.05). Analysis of the heart structures of the patients with CS using the Ganau method. The left ventricular configuration was divided according to the Ganau classification, as follows: normal left ventricular configuration (N): normal LVMI and RWT < 0.45; concentric remodeling (CR): normal LVMI and RWT ≥ 0.45; centripetal hypertrophy (CH): increased LVMI and RWT ≥ 0.45; eccentric hypertrophy (HE): increased LVMI and RWT < 0.45. The analysis showed that 22.4% of patients with CS had structural abnormalities of the heart, including 11.2% with centripetal remodeling, 5.6% with centripetal hypertrophy, and 5.6% with eccentric hypertrophy (Table 5 and Fig. 4).

**Table 5:** Comparison of cardiac structural and functional characteristics among etiological groups in patients with Cushing’s syndrome.

	Total	ACA	CD	EAS	P value
Myohemoglobin	31.20 (24.20, 45.70)	25.60 (22.30, 42.90)	31.20 (25.80, 45.70)	45.20 (30.50, 129.80)	0.109
Troponin	0.01 (0.01, 0.03)	0.01 (0.01, 0.02)^a^	0.01 (0.01, 0.03)^b^	0.05 (0.03, 0.10)^a, b^	0.003*
CK-MB	1.01 (0.62, 1.75)	0.97 (0.70, 1.60)^a^	0.72 (0.55, 1.33)^b^	2.26 (1.95, 3.31)^a, b^	0.007*
pro-BNP	450.0 (91.1, 756.0)	328.0 (80.7, 1450.0)	160.0 (54.3, 557.5)^a^	756.0 (552.0, 2020.0)^a^	0.018*
LAD	33.00 (31.00, 36.00)	33.00 (31.00, 35.00)	33.00 (30.00, 36.00)	38.00 (31.75, 45.25)	0.058
RVD	21.00 (20.00, 23.00)	21.00 (20.00, 23.00)^a^	22.00 (20.00, 23.00)	23.50 (21.50, 25.00)^a^	0.039*
IVST	10.00 (8.00, 10.00)	10.00 (8.00, 10.00)	9.00 (8.00, 10.00)^a^	10.50 (9.00, 12.75)^a^	0.030*
LVEDD	47.00 (43.00, 49.00)	45.00 (43.00, 48.50)^a^	47.00 (44.00, 49.00)	49.00 (46.50, 55.50)^a^	0.013*
LVPWT	9.00 (8.00, 10.00)	9.00 (8.00, 10.00)	9.00 (8.00, 10.00)	10.00 (9.00, 10.00)	0.078
EF	62.00 (60.00, 64.00)	62.00 (59.00, 64.00)	63.00 (60.00, 64.00)	64.00 (59.00, 69.00)	0.222
E/A ratio	0.78 (0.67, 1.24)	0.84 (0.70, 1.28)	0.78 (0.65, 1.12)	0.71 (0.68, 0.76)	0.121
LVMI	86.06 (73.73, 99.36)	84.87 (71.48, 98.85)^a^	86.03 (73.58, 98.57)^b^	111.47 (87.29, 146.06)^a, b^	0.016*
RWT	0.38 (0.36, 0.42)	0.38 (0.36, 0.42)	0.38 (0.36, 0.41)	0.40 (0.34, 0.43)	0.657

CK-MB, creatine kinase isoenzyme; pro-BNP, pro-brain natriuretic peptide; LAD, left atrial diameter; RVD, right ventricular diameter; IVST, interventricular septal thickness; LVEDD, left ventricular end-diastolic diameter; LVPWT, left ventricular posterior wall thickness; EF, ejection fraction; E/A ratio, the mitral early to late diastolic flow velocity ratio; LVMI, left ventricular mass index; RWT, relative wall thickness.

### Risk factors for Cushing's syndrome recurrence

All patients with CS caused by pituitary tumors and adrenal adenomas were followed up and the time of tumor recurrence was recorded. The median estimated time to relapse for all patients was 103 months and the mean time to relapse was 85 months, with an overall recurrence rate of 25.7%. Among them, the recurrence rate was 38.1% in the CD group and 8.7% in the ACA group.

**Figure 5:**
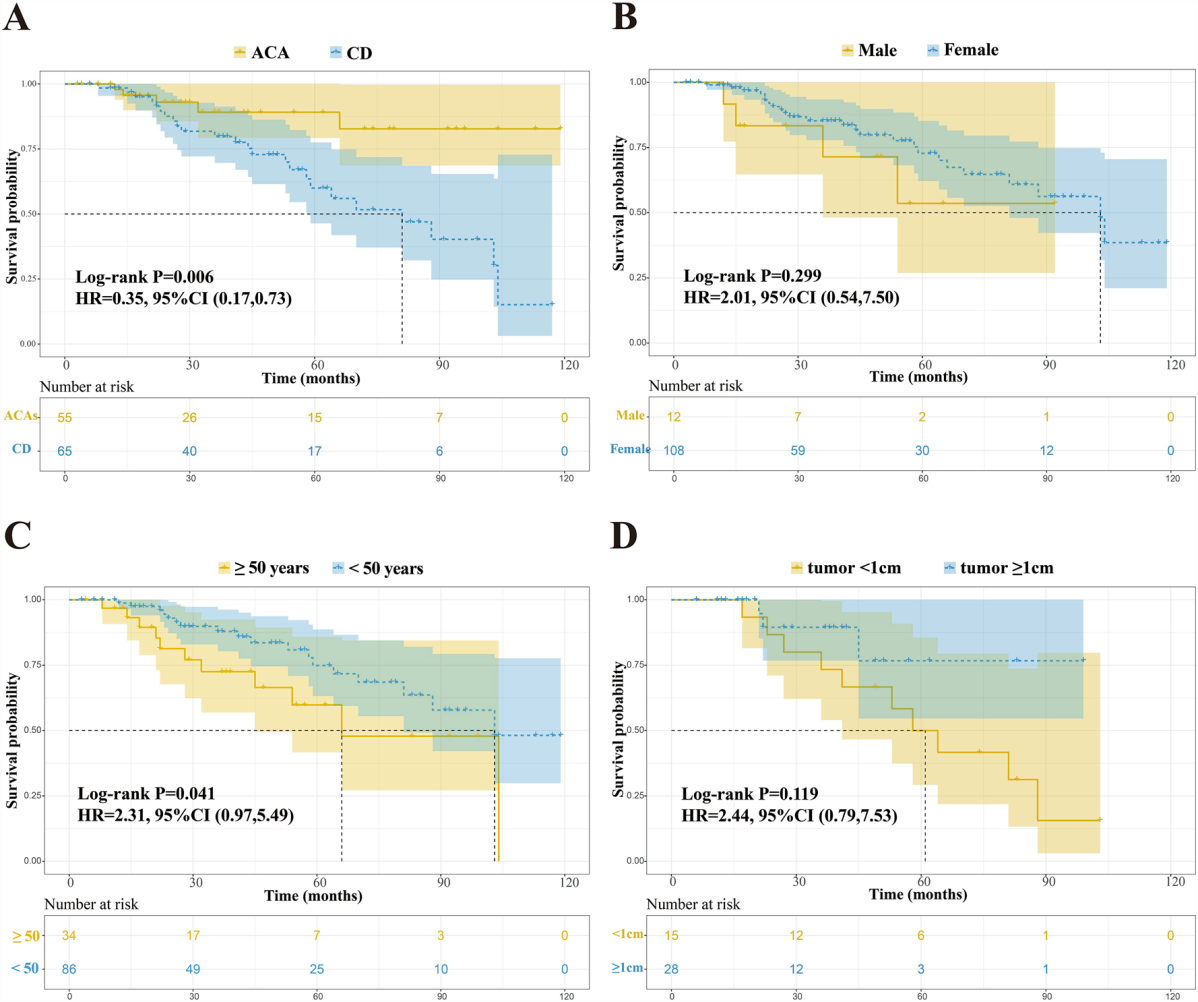
(**A**) The recurrence time in the adrenocortical adenoma (ACA) group was longer than that in the Cushing's disease (CD) group (hazard ratio [HR] = 0.35, P = 0.006. (**B**) Sex shows no significant effect on the recurrence time (P = 0.299). (**C**) The recurrence time in the < 50-years group was longer than that of the ≥ 50-years group (HR = 2.31, P = 0.041). (**D**) Among patients with Cushing's disease, the recurrence time of patients with tumors ≥ 1 cm was longer than that of patients with tumors < 1 cm group (HR = 2.44, P = 0.119).

The patients were grouped according to the patient sex and age and CS etiology and tumor volume. The Kaplan–Meier method was used to estimate the recurrence rate of patients at different times, and the log-rank method was used to examine the survival curve (Fig. 5).

The Cox proportional risk model was used to analyze the risk factors for CS tumor recurrence and Cox regression was used for univariate analysis. The following variables were included: etiology, sex, age, tumor volume in Cushing's disease, BMI, preoperative cortisol level, preoperative ACTH level, preoperative 24 h-UFC, postoperative cortisol level, and absolute values of neutrophil and eosinophil counts. The risk factors for CS recurrence were etiology (P = 0.006), age (P = 0.041), preoperative cortisol level (P = 0.021), postoperative cortisol level (P = 0.052), and absolute neutrophil count (P = 0.088). We included variables with P < 0.1 in the univariate analysis in the multivariate Cox regression. The results of the multivariate Cox regression analysis showed that etiology was an independent predictor of postoperative recurrence (P = 0.011). Age, preoperative and postoperative cortisol levels, and absolute neutrophil count were closely related to postoperative recurrence (Table 6).

**Table 6:** Univariate and multivariable results of Cox proportional hazards regression of factors affecting the recurrence of Cushing's syndrome.

	Univariable hazard ratio (95% CI)	P value	Multivariable hazard ratio (95% CI)	P value
Etiology				
CD	1.00		1.00	
ACA	0.35 (0.17, 0.73)	0.006*	0.29 (0.11, 0.76)	0.011*
Sex				
Female	1.00			
Male	2.01 (0.54, 7.50)	0.299		
Age				
< 50	1.00		1.00	
≥ 50	2.31 (0.97, 5.49)	0.041*	1.93 (0.91, 4.09)	0.088
Tumor size (CD group)				
≥ 1 cm	1.00			
< 1 cm	2.44 (0.79, 7.53)	0.119		
BMI	1.06 (0.93, 1.21)	0.386		
Preoperative cortisol	1.00 (1.00, 1.00)	0.021*	1.00 (1.00, 1.00)	0.275
Preoperative ACTH				
CD group	0.99 (0.95, 1.02)	0.435		
ACAs group	1.41 (0.92, 2.17)	0.115		
Preoperative 24H-UFC	1.00 (1.00, 1.00)	0.879		
Postoperative cortisol	1.00 (1.00, 1.00)	0.052*	1.00 (1.00, 1.00)	0.156
ANC	1.12 (0.98, 1.27)	0.088*	1.07 (0.92, 1.24)	0.406
AEC	0.03 (0.00, 225.99)	0.430		

CI, confidence interval; CD, Cushing's disease; ACA, adrenocortical adenoma; BMI, Body mass index; ACTH, adrenocorticotropin; 24H-UFC, 24-hour urinary free cortisol; ANC, absolute neutrophil count; AEC, absolute eosinophil count.

## Discussion

This retrospective study of 202 patients with CS is the first to analyze the effects of different etiologies on cardiac structural function and is the first to report etiology as an independent predictor of postoperative recurrence. Moreover, this is one of the largest studies to date to provide long-term follow-up data from a single institution.

It is well known that patients with CS usually exhibit typical clinical symptoms such as central obesity, facial acne, violaceous striae and hypertension^5,10–14^. However, unlike patients with CD and ACA, EAS usually presents with rapidly progressive severe Cushing's syndrome, such as refractory hypertension, severe electrolyte disturbances, and metabolic abnormalities^15–18^. Our study found that common symptoms among CS patients in the total population included hypertension (71.78%), central obesity (71.29%) and type 2 diabetes mellitus (35.15%). Patients in the EAS group showed more oedema (77.78%), infections (44.44%), fatigue (66.67%), and type 2 diabetes mellitus (66.67%) than patients in the CD and ACA groups. In contrast, patients in the CD and ACA groups tended to exhibit central obesity and violaceous striae. Notably, we found that 44.44% of patients in the EAS group had symptoms of infection, while only 5.74% of patients in the CD group and 4.23% of patients in the ACA group had symptoms of infection. Therefore, in patients with EAS, close monitoring of infections may be necessary to prevent the development of serious infections.

CS is often associated with electrolyte disturbances, and 41.9% of patients have hypokalemia. Compared with those with CD and ACA, patients with EAS are more prone to hypokalemia. The mechanisms underlying hypokalemia are not fully understood. Ulick et al. observed a relative deficiency in 11β-hydroxysteroid dehydrogenase activity in patients with CS and hypokalemia. Excess cortisol acted as a mineralcorticoid by binding to the mineralcorticoid receptor, leading to urinary potassium excretion and sodium retention, which further led to hypokalemia and hypertension^19^. Serum and urinary cortisol levels were negatively correlated with potassium levels (P < 0.05), consistent with the results reported by Linling et al.^6^. Ariadne et al. found that 16% of patients with CS had hypophosphatemia, with a negative correlation between 24 h-UFC levels and serum phosphate concentrations^7^. Hypophosphatemia was present in 15.0% of the patients in our study, including 15.8%, 18.7%, and 28.6% of patients in the ACA, CD, and EAS groups, respectively. Serum cortisol and phosphorus levels were negatively correlated (P < 0.05). Patients with CS can develop hypophosphatemia through increased urinary phosphate excretion or inhibition of intestinal phosphate absorption. Moreover, hypophosphatemia can aggravate fatigue symptoms in patients^20–22^.

Previous studies reported higher risks of myocardial infarction and heart failure among patients with CS compared with the general population, with an increased prevalence of left ventricular hypertrophy and centripetal remodeling often accompanied by diastolic filling disorders^8,9,23,24^. In our study, 22.4% of the patients had heart structural abnormalities, including 11.2% with centripetal remodeling, 5.6% with centripetal hypertrophy, and 5.6% with eccentric hypertrophy. The differences in cardiac structure and function indices due to different causes were statistically significant (P < 0.05). The most important ultrastructural abnormality in CS-induced cardiomyopathy is myocardial fibrosis, which mainly occurs due to the enhanced action of cortisol on angiotensin II^25^. Activation of mineralcorticoid and glucocorticoid receptors also contributes to its development. Vascular remodeling and increased vascular oxidative stress play important roles in the cardiovascular complications of CS^13^. Peter et al. showed that in the absence of dense myocardial fibrosis, patients with CS often have significant subclinical biventricular and left atrial systolic dysfunction and an increased left ventricular mass^9^. Structural and functional abnormalities of the myocardium occur in the early stages of CS; therefore, active screening for cardiac color ultrasound and cardiac function indicators are required to prevent heart failure.

Surgery is the best treatment for CS caused by pituitary tumors and adrenal adenomas, and 80%–90% of patients achieve initial remission. However, approximately one-quarter of the patients with initial remission experience recurrence^26^. The early detection and identification of patients at risk of recurrence will allow early treatment. Previous studies showed that postoperative cortisol levels < 2.0 µg/dL have excellent predictive value and that patients with plasma cortisol levels > 2.0 µg/dL have twice the risk of recurrence compared with patients with levels < 2.5 µg/dL^26–28^. Midnight salivary cortisol levels have also been used to identify patients at increased risk of relapse^29^. A recent study found that midnight salivary cortisol levels after surgery predicted recurrence in patients with 100% sensitivity and 98% specificity. The sensitivity and specificity of plasma cortisol for predicting recurrence were 93% and 74% respectively^30^. The median estimated time to relapse for all patients was 103 months, and the overall recurrence rate was 25.7%. The risk factors for CS recurrence included etiology, age, preoperative cortisol level, postoperative cortisol level, and absolute neutrophil count. To our knowledge, this is the first study to show that etiology is an independent predictor of postoperative recurrence. Age, preoperative and postoperative cortisol levels, and absolute neutrophil count were closely related to postoperative recurrence. The recurrence time in the ACA group was longer than that in the CD group. Sex had no significant effect on recurrence time. The recurrence time in the age group < 50 years was longer than that in the age group ≥ 50 years. In Cushing's disease, the recurrence time of tumors ≥ 1 cm was longer than that of tumors < 1 cm. Previous studies demonstrated a higher recurrence rate of macroadenomas than that of microadenomas in patients with Cushing's disease. However, a recent single-center study reported the opposite, consistent with our findings^31,32^.

This study was a single-center retrospective study. Due to the small number of patients with ectopic ACTH syndrome in clinical practice, the number of EAS cases included in the study was low, resulting in certain limitations in the analysis results. Further research with large sample sizes in multiple centers is needed in the future.

## Conclusions

In summary, we compared the clinical features of CS caused by pituitary, adrenal, and ectopic ACTH tumors and analyzed the risk factors for CS recurrence. We found that central obesity was the most common clinical symptom in the ACAs group, and hypertension was the most common clinical symptom in the CD and EAS groups. Hypokalemia was present in 41.9% of patients with CS and hypophosphatemia in 15.0%. CS may also affect the structural function of the heart, with 22.4% of patients with CS having structural cardiac abnormalities. The overall recurrence rate of CS due to pituitary and adrenal adenomas was 24.2%. Etiology is an independent predictor of tumor recurrence in patients with CS.

## Data Availability

The raw data supporting the conclusions of this article will be made available by the authors, without undue reservation. The datasets used or analyzed during the current study are available from the corresponding author on reasonable request.

## Abbreviations

CS: Cushing's syndrome
CD: Cushing's disease
ACA: Adrenocortical adenoma
ACTH: Adrenocorticotropic hormone
ANOVA: Analysis of variance
EAS: Ectopic ACTH syndrome
24H-UFC: 24-Hour urinary free cortisol
BMI: Body mass index
BSA: Body surface area
NLR: Neutrophil-to-lymphocyte ratio
LVM: Left ventricular mass
LVMI: LVM index
RWT: Relative wall thickness
CK-MB: Creatine kinase isoenzyme
pro-BNP: Pro-brain natriuretic peptide
N: Normal left ventricular configuration
CR: Concentric remodeling
CH: Centripetal hypertrophy
HE: Eccentric hypertrophy
HR: Hazard ratio
LAD: Left atrial diameter
RVD: Right ventricular diameter
IVST: Interventricular septal thickness
LVEDD: Left ventricular end-diastolic diameter
LVPWT: Left ventricular posterior wall thickness
EF: Ejection fraction
E/A ratio: The mitral early to late diastolic flow velocity ratio
CI: Confidence interval
K: Potassium
Na: Sodium
Cl: Chloride
Ca: Calcium
P: Phosphorus
ANC: Absolute neutrophil count
AEC: Absolute eosinophil count
